# Cardiac endothelial cell-derived exosomes induce specific regulatory B cells

**DOI:** 10.1038/srep07583

**Published:** 2014-12-23

**Authors:** Jiangping Song, Xiao Chen, Mangyuan Wang, Yong Xing, Zhe Zheng, Shengshou Hu

**Affiliations:** 1 State Key Laboratory of Cardiovascular Disease, Fuwai Hospital, National Center for Cardiovascular Diseases, Chinese Academy of Medical Sciences and Peking Union Medical College, 167A Beilishi Road, Xi Cheng District, Beijing. 100037, China

## Abstract

The mechanism of immune tolerance is to be further understood. The present study aims to investigate the role of the Cardiac endothelial cell (CEC)-derived exosomes in the induction of regulatory B cells. In this study, CECs were isolated from the mouse heart. Exosomes were purified from the culture supernatant of the primary endothelial cells. The suppressor functions of the regulatory B cells were determined by flow cytometry. The results showed that the CEC-derived exosomes carried integrin αvβ6. Exposure to lipopolysaccharide (LPS) induced B cells to express the latent transforming growth factor (TGF)-β, the latter was converted to the active form, TGF-β, by the exosome-derived αvβ6. The B cells released TGF-β in response to re-exposure to the exosomes in the culture, which suppressed the effector T cell proliferation. We conclude that CEC-derived exosomes have the capacity to induce B cells with immune suppressor functions.

The induction of immune tolerance to alloantigens is a potential approach to inhibit the alloantigen-related immune responses, such as allograft rejection. The CD4^+^ CD25^+^ Foxp3^+^ regulatory T cells (Treg) are one of the major immune regulatory cells. Published data indicate that the secretion of suppressive cytokines by Tregs, such as IL-10, transforming growth factor-β (TGF-β), and IL-35, is associated with the immunosuppressive functions of Treg^1^. Recent studies suggest that a fraction of B cell also has immune regulatory functions; these B cells are designated regulatory B cells (Breg)^2^. Similar to Tregs, Bregs also express TGF-β^3^ or IL-10^4^. However, the generation of Bregs is not fully understood yet.

After synthesis, TGF-β exists as a latent form, the latent TGF-β (LTGFβ). A latency associated peptide (LAP) is attached to TGF-β to form a complex that prevents the TGF-β from interacting with other molecules. To activate LTGFβ, the LAP has to be removed from the complexes, which can be carried out by many proteases, such as plasmin, β6 integrin, αV integrin, β8 integrin^5^,^6^. We have found that intestinal epithelial cell-derived exosomes carry αvβ6, which can induce tolerogenic dendritic cells (DC)^6^. Based on the information above, we hypothesize that the CEC-derived exosomes carry αvβ6 to contribute to the establishment of immune tolerance. In this study, we cultured primary CECs, purified exosomes from the culture supernatant. The exosomes induced the TGF-β^+^ B cells. These TGF-β^+^ B cells released TGF-β in the culture upon re-exposure to the exosomes.

## Results

### Activation of TLR4 increases integrin αvβ6 in CEC-derived exosomes

**Figure 1:**
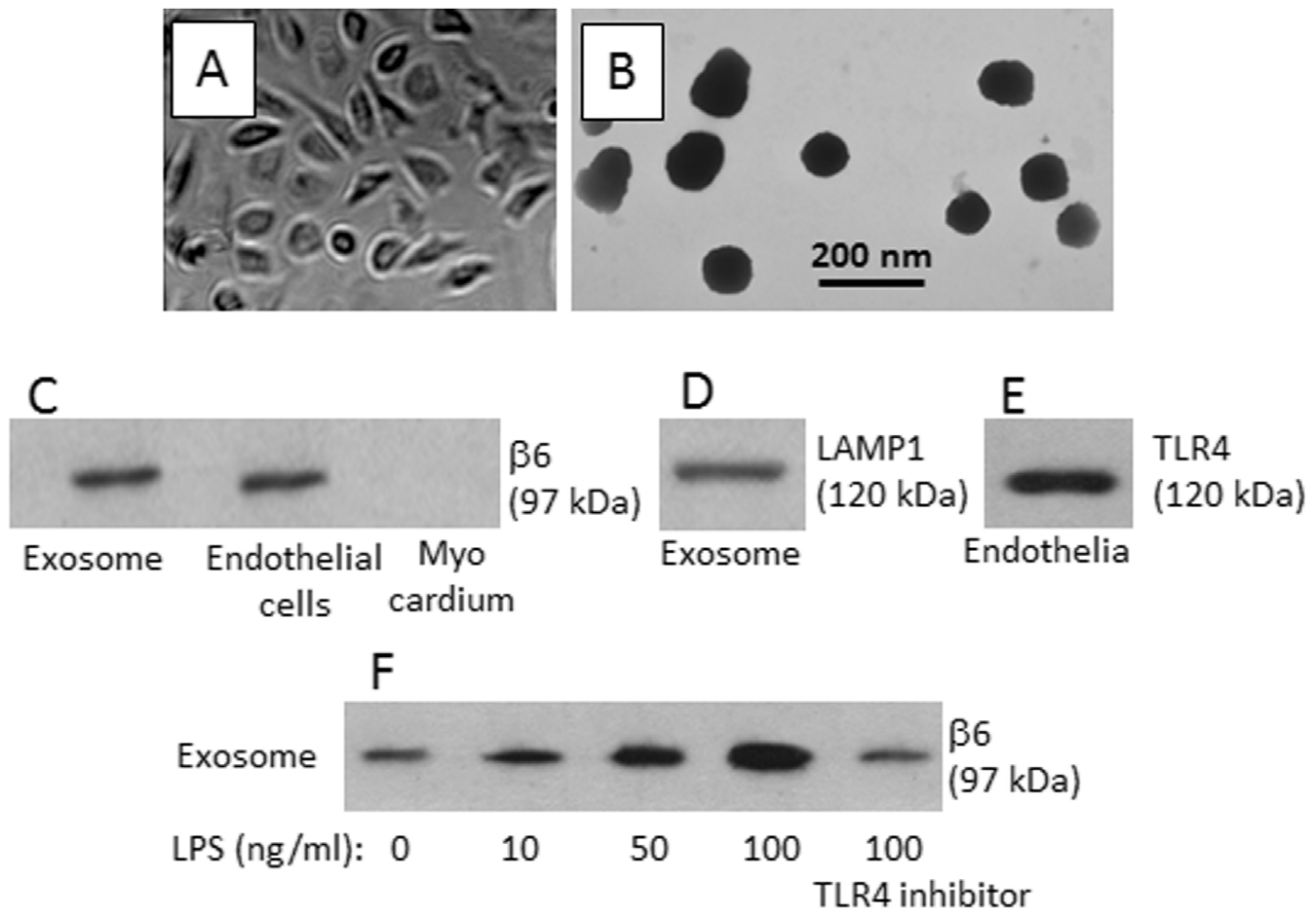
Cardiac endothelial cell (CEC)-derived exosomes contain integrin αvβ6. The CEC-derived exosomes were generated from the mouse hearts as described in the text. (A–B), light microscope images show the primary culture of mouse CECs on day 10 (A). Original magnification: ×200. (B), a representative electron microscope image shows the CEC-derived exosomes (original magnification: ×300,000). (C–H), immune blots indicate the proteins of β6 (C) in the extracts of exosomes, CECs and myocardium, respectively. (D): LAMP1 (a marker of exosomes). (E): TLR4 in the endothelial cell extracts. (F), the cardiac endothelial cells were exposed to LPS in the culture for 48 h. Exosomes were purified from the culture supernatant. The immune blots show the contents of β6 in the extracts of exosomes. TLR4 inhibitor (TAK242): 1 μM. The data are a representative of 3 independent experiments.

Inspired by published data that dendritic cell-derived exosomes have immune tolerogenic features^7^, we prepared CECs (Fig. 1A); exosomes were purified from the cell culture supernatant (Fig. 1B). Integrin αvβ6 was detected in the CECs and exosomes, but not in the cardiac myocardium (Fig. 1C). LAMP1 (a marker of exosomes) was detected in the extracts of the exosomes (D). Toll-like receptor (TLR)4 was detected in the endothelial cells (Fig. 1E). The endothelial cells were exposed to LPS in the culture for 48 h, which enhanced the levels of αvβ6 significantly in an LPS dose-dependent manner (Fig. 1F). To confirm the results, TLR4 inhibitor was added to the culture. Indeed, the LPS-induced increases in αvβ6 were abolished (Fig. 1F). The data indicate that the CEC-derived exosomes carry αvβ6. Exposure to LPS increases the levels of αvβ6 in the exosomes.

### CEC-derived exosomes convert latent TGF-β in B cells

**Figure 2:**
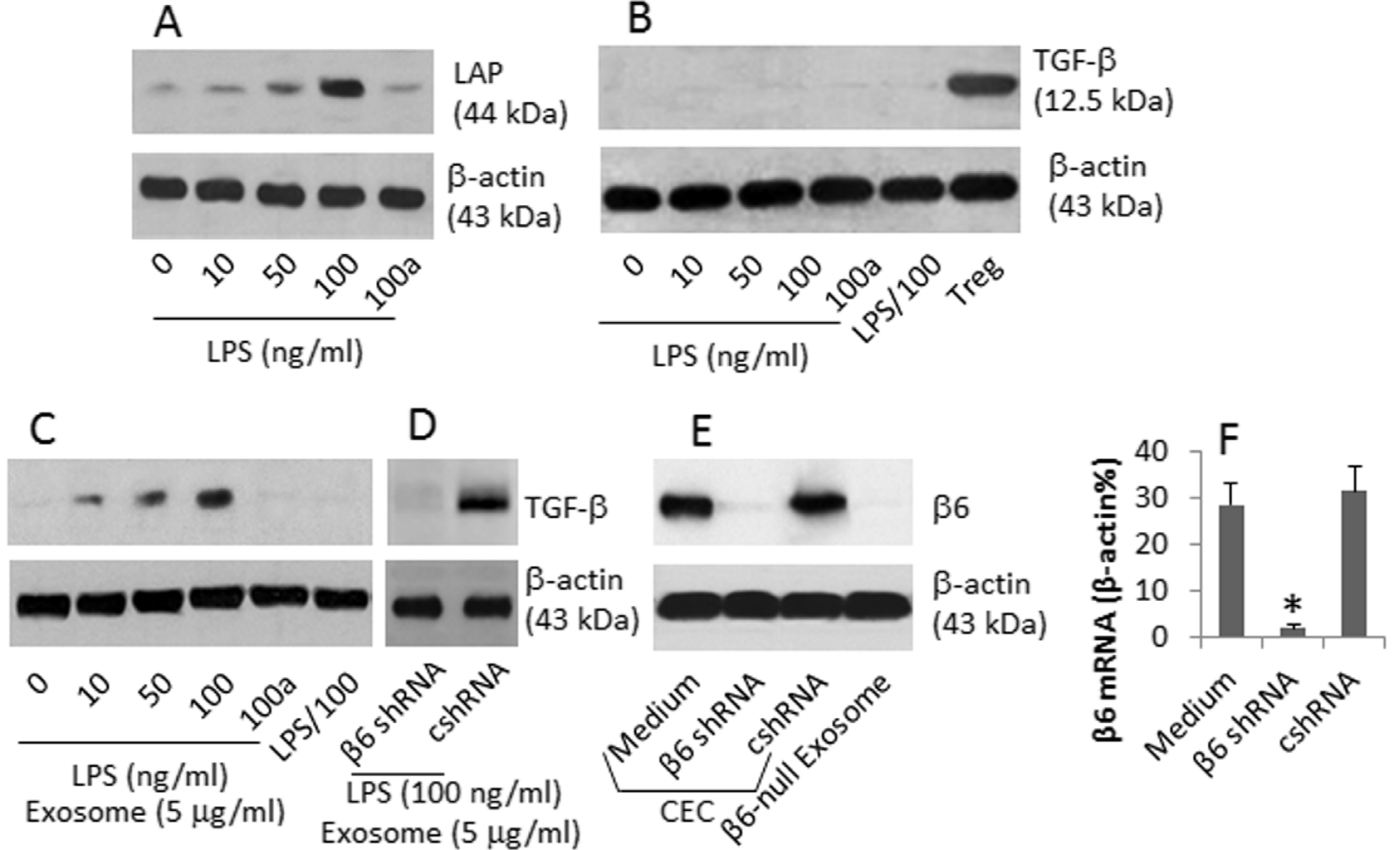
CEC-derived exosomes induce TGF-β in B cells. CD19^+^ IL-7R^+^ CD45^+^ B cells were cultured in the presence of LPS or/and exosomes, and an anti-CD40 antibody (1 μg/ml) for 7 days. The cell extracts were analyzed by Western blotting. The immune blots show the levels of LAP (A) and TGF-β (B, C). a: B cells were treated with TLR4 inhibitor (TAK242, 1 μM). Treg: The proteins were extracted from the CD4^+^ CD25^+^ CD127^−^ Tregs (from the spleen; isolated by MACS; using as a positive control). (D), the immune blots indicate the levels of TGF-β in the B cells treated with exosomes from CECs treated with β6 shRNA or control shRNA (cshRNA). (E), the immune blots show the protein of β6 in CECs or β6-null exosomes. (F), the bars indicate the mRNA levels of β6 in CECs (mean ± SD. *, p < 0.01, compared to medium group). The data are a representative of 3 independent experiments.

The results of Fig. 1 suggest that the αvβ6-laden exosomes can be released out of the endothelial cells; the exosomes may be endocytosed by immune cells, such as the antigen presenting cells. B cells are one type of the antigen presenting cells. Next, we isolated naïve B cells from the bone marrow and cultured in the presence of the exosomes or/and LPS for 7 days, and then the expression of the immune regulatory molecules of TGF-β and the latent associated proteins (LAP) by the B cells were assessed. The results showed that the exposure to LPS increased the levels of LAP (Fig. 2A), but not TGF-β (Fig. 2B), in B cells. Exposure to exosomes alone also did not increase TGF-β (Fig. 2C); however, exposure to both LPS and exosomes markedly increased the levels of TGF-β in the B cells, which was abolished by the addition of TLR4 inhibitor to the culture (Fig. 2C) or exposure to exosomes produced by the β6-null CEC (Fig. 2D–F).

### Phenotypes of the TGF-β^+^ B cells generated by the CEC-derived exosomes

**Figure 3:**
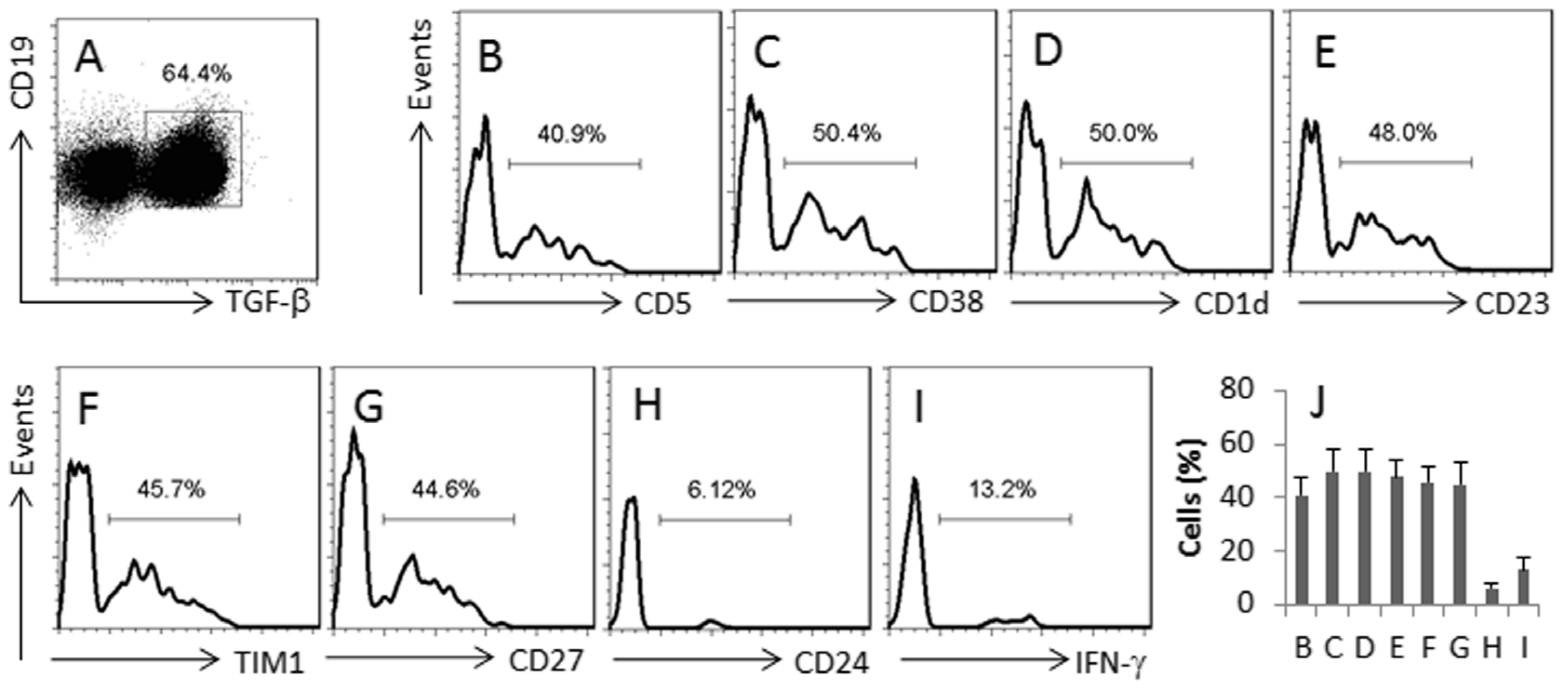
Phenotypes of the TGF-β^+^ B cells. The TGF-β^+^ B cells were generated by exposing CD19^+^ IL-7R^+^ CD45^+^ B cells to the CEC-derived exosomes and LPS. (A), the gated dot plots show the frequency of CD19^+^ TGF-β^+^ B cells. (B–I), the histograms indicate the phenotypes (denoted below each histogram) of the gated cells in panel (A. J), the bars indicate the summarized cell population of (B–I) (mean ± SD). The data represent 3 separate experiments.

Following the same procedures above, we treated naïve B cells with the CEC-derived exosomes and LPS in the culture for 7 days. The cells were analyzed by flow cytometry. About 64.4% B cells showed TGF-β^+^ (Fig. 3A). Among the TGF-β^+^ cells, high frequency of CD5^+^, CD38^+^, CD1d^+^, TIM1^+^, CD23^+^, CD27^+^ cells were detected, and low frequency of IFN-γ^+^ and CD24^+^ cells were also detected (Fig. 3B–J).

### CEC-derived exosomes induce TGF-β^+^ B cells

**Figure 4:**
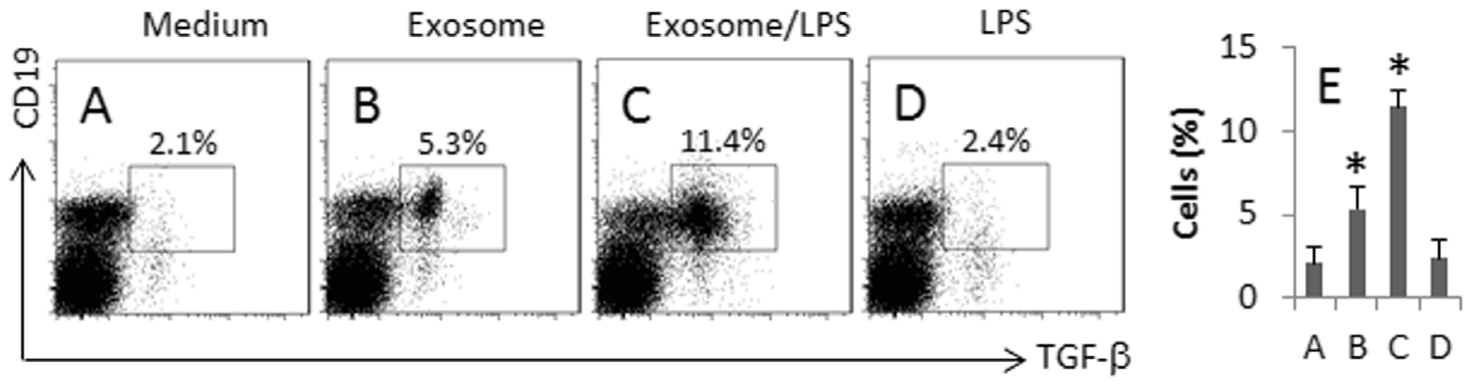
Induction of the expression of TGF-β in B cells. The TGF-β^+^ B cells were generated as described in the text. The treatment is denoted above each subpanel. The cells were analyzed by flow cytometry. (A–D), the gated dot plots show the frequency of TGF-β^+^ B cells. E, the bars indicate the summarized data of (A–D). Exosome = 5 μg/ml. LPS = 100 ng/ml. The data of bars are presented as mean ± SD. *, p < 0.01, compared to group A. The data are a representative of 3 independent experiments.

We generated the exosome-specific TGF-β^+^ B cells by exposing naïve B cells to the exosomes or/and LPS for 7 days. As shown by flow cytometry data, not much TGF-β+ B cells were induced when the cells were cultured in medium alone (Fig. 4A, 4E); exposure to the exosomes moderately increased the TGF-β^+^ B cells (Fig. 4B, 4E), which was further increased by adding the exosomes and LPS to the culture (Fig. 4C, 4E). The presence of LPS in the culture alone did not induce TGF-β in the B cells (Fig. 4D, 4E). The results suggest that the CEC-derived exosomes are capable of inducing TGF-β expression in B cells, which can be promoted by the presence of LPS in the culture.

### Exposure to CEC-derived exosomes induces TGF-β release from the TGF-β^+^ B cells

**Figure 5:**
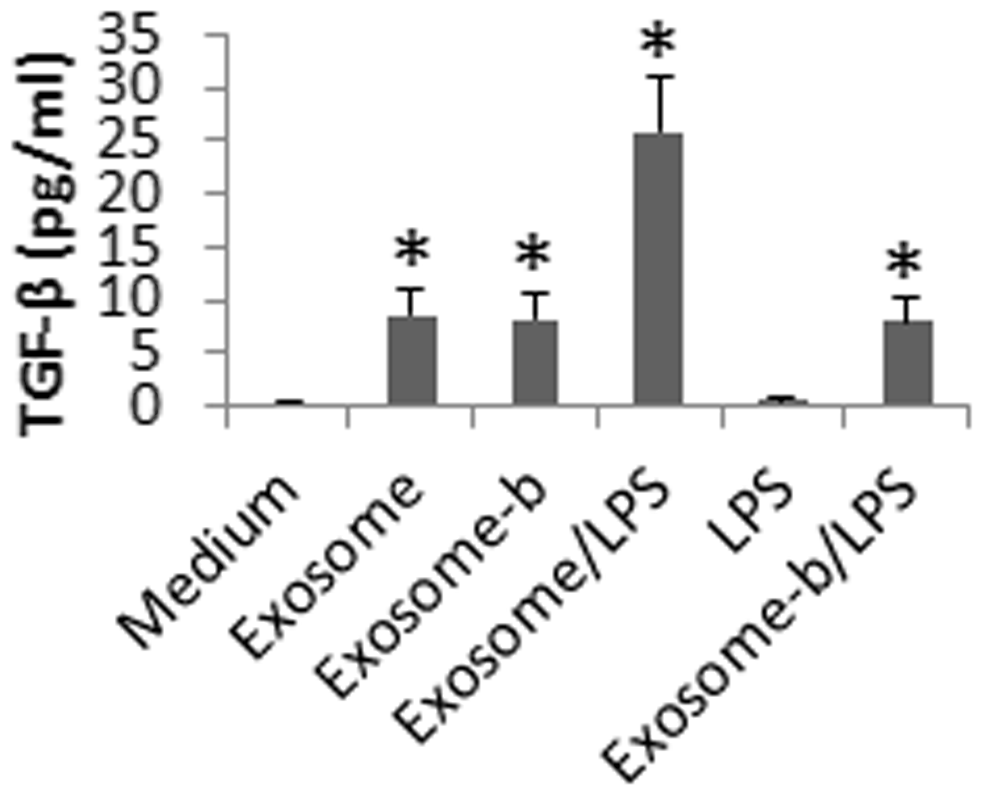
Exposure to CEC-derived exosomes induces TGF-β release. The TGF-β^+^ B cells were exposed to the CEC-derived exosomes or and LPS in the culture overnight as denoted on the X axis. Exosome-b: The β6-null exosomes. The culture supernatant was analyzed by ELISA. The bars indicate the TGF-β levels in the culture supernatant (mean ± SD; *, p < 0.01, compared to the medium group). Exosome (or exosomes-b) = 5 μg/ml. LPS = 100 ng/ml. The data are a representative of 3 independent experiments.

We induced the TGF-β^+^ B cells as indicated above; the cells were re-stimulated with exosomes or/and LPS. The supernatant was analyzed by ELISA. As shown by Fig. 5, the exposure to exosomes in the culture induced the release of TGF-β, which was further increased by the addition of LPS. Exposure to LPS alone did not induce the release of TGF-β. To elucidate if the αvβ6 carried by the exosomes played any roles in the TGF-β release from the B cells, we generated αvβ6-null exosomes. These αvβ6-null exosomes still induced the release of TGF-β, which was not further increased by the addition of LPS. The results suggest that re-exposure to the CEC-derived exosomes can induce the release of TGF-β from the B cells. Although αvβ6 is required in the generation of TGF-β^+^ B cells, the αvβ6 in the exosomes does not play a critical role in the TGF-β release from the TGF-β^+^ B cells. LPS can promote the release of TGF-β from the B cells in synergy with αvβ6.

### Immune suppressor function of the CEC-derived exosome-induced TGF-β^+^ B cells

**Figure 6:**
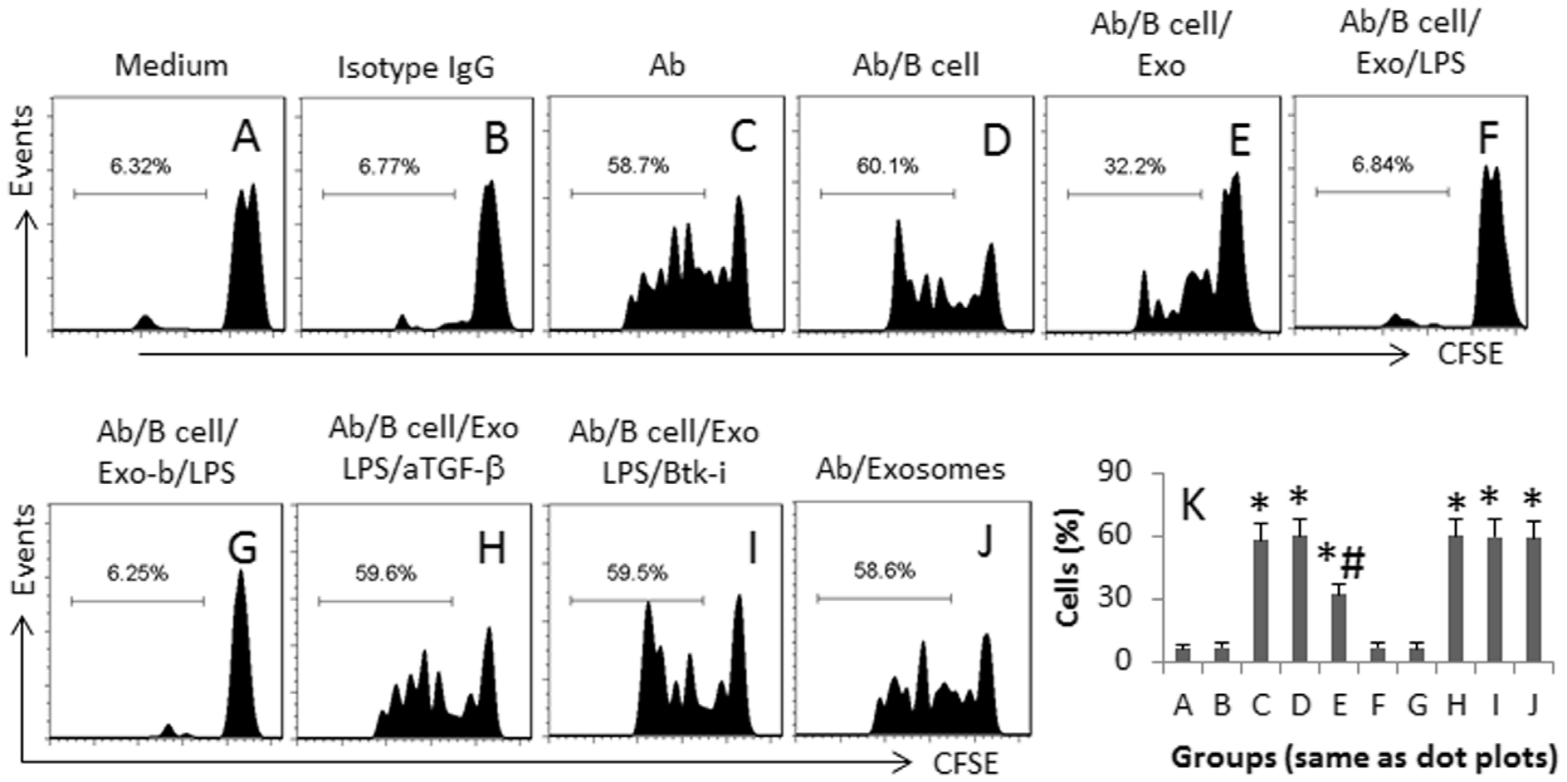
Specific activation of TGF-β^+^ B cells suppresses effector T cell proliferation. TGF-β^+^ B cells were generated as described in the text and cultured with CD4^+^ CD25^−^ T effector cells (Teff cell, labeled with CFSE) (B cell: T cell = 1:1) in the presence of anti-CD3/CD28 antibodies (Ab) with or without the presence of LPS or/and exosomes in the culture for 3 days. The additional treatments are denoted above each sub-panel. The cells were analyzed by flow cytometry. (A–J), the histograms show the frequency of proliferating Teff cells. Exo: Exosomes (5 μg/ml). LPS: 100 ng/ml. aTGF-β: Neutralizing anti-TGF-β antibody (10 μg/ml). Etk-i: Etk inhibitor (10 nM). (K), the bars indicate the summarized data of (A–J) (mean ± SD. *, p < 0.01, compared to group A. #, p < 0.01, compared to group D). The data are a representative of 3 independent experiments.

CD4^+^ T cells play a critical role in the skewed immune response, such as the transplantation rejection^8^, which may be inhibited by the TGF-β^+^ T cells^9^. To observe the immune suppressor function of the exosome-induced TGF-β^+^ B cells, we generated the TGF-β^+^ B cells using the above procedures and isolated CD4^+^ CD25^−^ T effector cells from the spleen. The T cells (labeled with CFSE) and B cells were cultured in the presence of anti-CD3/CD28 antibodies in the presence of exosomes or/and LPS for 3 days. The cells were then analyzed by flow cytometry. The results showed that after stimulating by anti-CD3/CD28, the T cells proliferated markedly (Fig. 6A–C). The presence of the B cells did not suppress the T cell proliferation (Fig. 6D). Considering an activator might be needed for the B cell activation to release TGF-β, we added exosomes to the culture, which partially suppressed the proliferation (Fig. 6E), and was significantly suppressed in the presence of both exosomes and LPS (Fig. 6F). To elucidate if the suppression was associated with the αvβ6 carried by the exosomes, we generated the αvβ6-null exosomes, which still showed the suppressor functions (Fig. 6G). The addition of a neutralizing anti-TGF-β antibody (Fig. 6H) or Etk inhibitor (I) to the culture efficiently inhibited the T cell proliferation. Exosomes alone did not show an inhibitory effect on the T cell proliferation (J). The summarized data of T cell proliferation are presented in Fig. 6K. The data indicate that the CEC-derived exosome-induced TGF-β^+^ B cells can be activated by the exosomes to suppress effector T cell activities, which can be strengthened by LPS.

## Discussion

The components of the endothelial cells may save as specific antigens to initiate a specific immune response to induce antigen specific immune reactions, such as to induce allograft rejection^10^ or other immune responses. Thus, to create an alloantigen specific immune tolerance is expected to improve the survival of the allograft or ameliorate the alloantigen-induced immune response. The present data indicate that CEC-derived exosomes carry integrin αvβ6. After exposure to the exosomes, B cells differentiate into TGF-β^+^ B cells. The TGF-β^+^ B cells can be activated by re-exposure to the exosomes to release TGF-β into the culture supernatant and suppress effector T cell proliferation.

TGF-β is one of the major immune regulatory molecules^11^. TGF-β-expressing T cells and B cells can be Tregs or Bregs. Thus, the TGF-β^+^ B cells, we observed in the present study, can be Bregs. The TGF-β^+^ B cells can be activated by exposure to exosomes, not by the bovine serum in the medium. The fact demonstrates that the TGF-β^+^ B cells, generated by the CEC-derived exosomes, are a kind of “exosome” antigen-specific TGF-β^+^ B cells. There are a number of components in the exosomes, the specific antigens induced the antigen-specific TGF-β^+^ B cells have not been specified in the present experiments and need to be elucidated in the future studies.

The data show that the CEC-derived exosomes carry αvβ6. This is in line with previous studies, such as Chen et al reports that intestinal epithelial cell-derived exosomes also carry αvβ6. αvβ6 is described by the early studies that can convert LTGFβ^12^ and followed by many others^13^,^14^. Thus, αvβ6 is an important molecule in the development of TGF-β^+^ cells with a premise that the cells produce LTGFβ. Although naïve B cells do not produce detectable LTGFβ, after exposure to LPS, the expression of LTGFβ is increased markedly as we observed in the present study. The data suggest that concurrent exposure to both αvβ6 and LPS may generate new immune regulatory cells. The inference is supported by our previous studies^6^ and others^15^,^16^.

CD4^+^ effector T cells are one of the major immune cells involving in a number of immune responses. In addition to the beneficial functions, CD4^+^ effector T cells act as inflammatory cells in the induction of immune inflammation in the body. Thus, to suppress the activities of CD4^+^ effector T cells to given extents has therapeutic effect on immune disorders. The regulatory cells, including Tregs and Bregs, can suppress immune inflammation associating with CD4^+^ effector T cells^16^,^17^. It is suggested that the treatment with Tregs prevents chronic rejection of heart allografts^18^ and inhibits intestinal inflammation^19^; and B cells also play a critical role in the complex immunoregulatory network in organ transplantation^20^. Our data are in line with those previous studies by showing that the TGF-β^+^ B cells suppress the effector T cell proliferation. It is noteworthy that the TGF-β^+^ B cells can be activated by exposure to the exosomes, and the activation can be strengthened by the addition of LPS to the culture.

Different results about the production of TGF-β by B cells in response to LPS has been reported. Parekh et al indicate that LPS can induce TGF-β production by B cells in the culture^21^; our data show that the exposure to LPS in the culture only induces the expression of LTGFβ; the latter needs to be activated by the integrin αvβ6. Such a discordance may be because the LPS concentrations using in our experiments are different from that of Parekh et al; the highest concentration of LPS is 0.1 μg/ml in our study while Parekh's LPS concentration is 20 μg/ml. TGF-β is a latent form after synthesis, it requires being activated prior to obtaining its biological activities^22^; the requirement might be met in the environment with relative high levels of LPS^21^. The inference is supported by published data that LPS can increase the expression of matrix metalloproteinase^23^; the latter can activate TGF-β^24^, which still needs to be further investigated with B cells in the future studies.

In summary, the present data indicate that the CEC-derived exosomes carry αvβ6; the latter converts LTGFβ to TGF-β in B cells. The generated TGF-β^+^ B cells can be activated by re-exposure to the exosomes to release TGF-β, and suppress effector T cell proliferation.

## Methods

### Reagents

The Btk inhibitor, PCI-32765, was purchased from MedChem Express (Shanghai, China). Lipopolysaccharide (LPS) was purchased from Sigma Aldrich (Beijing, China). Antibodies of β6 (H-110), LAMP1 (E-5), TLR4 (25), TGF-β (V), IL-10 (NYRmIL-10), LAP (T-17) were purchased from Santa Cruz Biotechnology (Shanghai, China). The ELISA kit of TGF-β and neutralizing anti-TGF-β (9016) antibody was purchased from R&D Systems (Shanghai, China). The antibodies for flow cytometry were purchased from BD Bioscience (Shanghai, China). The immune cell isolation kits were purchased from Miltenyi Biotech (Beijing, China). The reagents for qRT-PCR and Western blotting were purchased from Invitrogen (Beijing, China).

### Mice

C57BL/6 mice were purchased from the Beijing Experimental Animal Center (Beijing, China). The mice were maintained in a pathogen free environment. The animal experimental procedures were approved by the Animal Ethic Committee at Experimental center of Beijing Fuwai Hospital in accordance to the guidelines.

### Isolation of CECs

CECs were isolated from C57BL/6 mice. The hearts were excised, rinsed with phosphate buffered saline (PBS) and cut into small pieces (2 × 2 × 2 mm). The tissue fragments were transferred to DMEM and incubated for 45–60 min at 37°C with mild shaking. The clumps of cells were dispersed by forcing through a sterile 18 G needle. The resulting tissue slurry was filtered through a 40-μm pore-size cell strainer and the single-cell suspension was washed in DMEM with centrifugation at 400 × g for 5 min. The cell pellet was re-suspended in DMEM. The CECs were isolated with magnetic beads coated with an anti-CD31 antibody following the manufacturer's instructions. The isolated CECs were cultured in DMEM supplemented with 10% fetal bovine serum, 100 U/ml penicillin, 0.1 mg/ml streptomycin and 2 mM L-glutamine. The culture medium was changed in every 3 days. The CEC reached confluence about 9 days and used for further experiments.

### Isolation of exosomes

The CECs were cultured with no-serum culture medium overnight. Exosomes were purified from the culture supernatant of CEC according to our established procedures that were published elsewhere^6^. Briefly, CEC culture supernatant was centrifuged at 1000 g and 8,000 g to eliminate cell debris, and subsequently centrifuged at 60,000 g. The pellet was then washed with PBS and pelleted again at 100,000 g. Isolated exosomes were resuspended in PBS and filtered twice through 0.22-μm filters. Sample exosomes were processed for electron microscopy imaging with our established procedures^6^.

### Western blotting

Cells were lysed in RIPA buffer (25 mM Tris, pH 7.6, 150 mM NaCl, 1% Nonidet P-40, 1% sodium deoxycholate, and 0.1% sodium dodecyl sulfate) containing a protease-inhibitor cocktail on ice for 30 min. The cell lysates were centrifuged at 12,000 g for 15 min at 4°C. Proteins were fractioned by 10% SDS–PAGE and transferred to polyvinylidene difluoride membranes. The membranes were blocked with 5% non-fat milk in Tris-buffered saline with Tween 20 (TBST), followed by incubation with the primary antibodies (0.5–1 μg/ml) overnight at 4°C. After 3 washes in TBST, membranes were exposed to horseradish peroxidase–conjugated secondary antibody (1:1000) for 1 h at room temperature. Proteins were detected by enhanced chemiluminescence (ECL) and exposed to X-ray films. β-actin was used as a loading control.

### Flow cytometry

Cells were collected and analyzed by flow cytometry with our established procedures^25^. Briefly, in the surface staining, cells were blocked with 1% bovine serum albumin (BSA) for 30 min, incubated with fluorochrome-labeled antibodies (indicated in the figures) for 30 min on ice. In the case of together with the intracellular staining, after the surface staining, cells were fixed with 2% paraformaldehyde containing 0.1% Triton X-100 for 2 h; then stained with fluorochrome-labeled antibodies. Phorbol 12-myristate 13-acetate (PMA, 100 ng/ml), ionomycin (500 ng/ml), and brefeldin A (10 μg/ml) were added to the medium for the last 4 hours of culture. After washing with PBS, the cells were analyzed with a flow cytometer (FACSCanto II, BD Bioscience). The data were analyzed with the software FlowJo.

### Enzyme-linked immunosorbent assay (ELISA)

The levels of TGF-β in culture supernatant were determined using a commercial reagent kit following the manufacturer's instructions.

### Generation of TGF-β^+^ B cells

CD19^+^ IL-7 receptor^+^ CD45^+^ B cells were isolated from the mouse bone marrow by magnetic cell sorting (MACS). The cells were cultured in RPMI 1640 medium supplemented with 10% fetal bovine serum, 100 U/ml penicillin, 0.1 mg/ml streptomycin, 2 mM L-glutamine, 1 μg/ml anti-CD40 antibody, 100 ng/ml LPS and 5 μg/ml exosomes for 2–3 rounds (one round was 3 days). After 3-round cultures, the TGF-β^+^ B cells were more than 80% as assessed by flow cytometry.

### Assessment of TGF-β^+^ B cell immune suppressor functions

CD4^+^ CD25^−^ T effector cells (Teff cells) were isolated from the mouse spleen with a commercial reagent kit (purity was greater than 98%) by MACS. The Teff cells were stained with Carboxyfluorescein succinimidyl ester (CFSE), cultured with the TGF-β^+^ B cells at a ratio of 1:1 for 3 days in the presence of the exosomes (5 μg/ml) or/and LPS (100 ng/ml). The cells were analyzed by flow cytometry.

### Isolation of naïve B cells from the bone marrows

The femur bones were excised from C57BL/6 mice; the bone marrows were flushed with culture medium. The red blood cells were lysed with a lysis buffer. The bone marrow cells were incubated with magnetic bead-conjugated anti-CD19 antibody for 30 min on ice. The cell suspension was then allowed to run through a MACS column (Miltenyi Biotec) to allow for the retention of CD19^+^ cells in the column. After elution, the CD19^+^ cells were further incubated with magnetic bead-conjugated anti-CD45 and anti-CD127 (IL-7 receptor α chain) antibodies for 30 min on ice. The cell suspension was then allowed to run through a MACS column to allow for the retention of CD45^+^ and CD127^+^ cells in the column. The cells were eluted from the column and checked by flow cytometry; the purity of the cells was greater than 96% (checked by flow cytometry) and to be used in further experiments.

### Preparation of the αvβ6-null exosomes

The isolated CECs were treated with a β6 shRNA reagent kit following the manufacturer's instructions. Briefly, in a 12-well plate, when the CEC reached 50% confluence, the cells were tranduced by lentivirus carrying β6 shRNA or control shRNA at a multiplicity of infection (MOI) of 50. Cells were harvested at 72 hours after infection and the knockdown efficiency of β6 was evaluated by quantitative real-time RT-PCR and western blot analysis. The β6-null CEC were cultured to generate exosomes in the same procedures as described above.

### Statistics

The data are presented as means ± SD. ANOVA was used to test differences between groups. A p < 0.05 was set as a significant criterion.

## Author Contributions

J.S., X.C., M.W. and Y.X. performed experiments, analyzed data and reviewed the manuscript. Z.Z. and S.H. designed the project, supervised the experiments and wrote the paper.

## References

[b1] Ferrer I. R., Hester J., Bushell A. & Wood K. J. Induction of transplantation tolerance through regulatory cells: from mice to men. Immunol Rev 258, 102–116 (2014). 24517428 10.1111/imr.12158

[b2] Bird L. Immunometabolism: Regulatory B cells weigh in. Nat Rev Immunol 14, 6–7 (2014). 24336103 10.1038/nri3588

[b3] Lee-Chang C. *et al.* Inhibition of Breast Cancer Metastasis by Resveratrol-Mediated Inactivation of Tumor-Evoked Regulatory B Cells. J Immunol 191, 4141–4151 (2013). 24043896 10.4049/jimmunol.1300606 PMC3795852

[b4] Liu Y. *et al.* Role of IL-10-producing regulatory B cells in control of cerebral malaria in Plasmodium berghei infected mice. Eur J Immunol. 43, 2907–2918 (2013). 23893352 10.1002/eji.201343512

[b5] Li J. *et al.* The Role of Integrin alpha v beta 8 in Neonatal Hypoxic-Ischemic Brain Injury. Neurotox Res 17, 406–417 (2010). 19771486 10.1007/s12640-009-9117-y PMC2847421

[b6] Chen X. *et al.* Intestinal epithelial cell-derived integrin avβ6 plays an important role in the induction of regulatory T cells and inhibits an antigen-specific Th2 response. J Leukoc Biol. 90, 751–9 (2011). 21724807 10.1189/jlb.1210696

[b7] Li X. *et al.* Tolerance Induction by Exosomes from Immature Dendritic Cells and Rapamycin in a Mouse Cardiac Allograft Model. PLoS ONE 7, e44045 (2012). 22952868 10.1371/journal.pone.0044045 PMC3430614

[b8] Pietra B. A., Wiseman A., Bolwerk A., Rizeq M. & Gill R. G. CD4 T cell−mediated cardiac allograft rejection requires donor but not host MHC class II. J Clin Invest 106, 1003–1010 (2000). 11032860 10.1172/JCI10467 PMC314344

[b9] Zheng S. G. *et al.* Transfer of regulatory T cells generated ex vivo modifies graft rejection through induction of tolerogenic CD4+CD25+ cells in the recipient. Int Immunol 18, 279–289 (2006). 16415106 10.1093/intimm/dxh368

[b10] Wehner J. R. & Baldwin W. M. I. Cardiac allograft vasculopathy: do adipocytes bridge alloimmune and metabolic risk factors? Curr Opin Organ Transplant. 15, 639–44 (2010). 20689436 10.1097/MOT.0b013e32833deaee

[b11] Oh S. A. & Li M. O. TGF-beta: Guardian of T Cell Function. J Immunol 191, 3973–3979 (2013). 24098055 10.4049/jimmunol.1301843 PMC3856438

[b12] Munger J. S. *et al.* A Mechanism for Regulating Pulmonary Inflammation and Fibrosis: The Integrin alpha v beta 6 Binds and Activates Latent TGF beta. Cell 96, 319–328 (1999). 10025398 10.1016/s0092-8674(00)80545-0

[b13] Shi M. *et al.* Latent TGF-[bgr] structure and activation. Nature 474, 343–349 (2011). 21677751 10.1038/nature10152 PMC4717672

[b14] Wipff P. J. & Hinz B. Integrins and the activation of latent transforming growth factor beta 1-An intimate relationship. Eur J Cell Biol. 87, 601–615 (2008). 18342983 10.1016/j.ejcb.2008.01.012

[b15] Wu X. M. *et al.* Integrin alphavbeta6 is involved in measles protein-induced airway immune suppression. Cytokine 59, 59–64 (2012). 22579114 10.1016/j.cyto.2012.04.005

[b16] Yang S. B. *et al.* Integrin alphavbeta6 promotes tumor tolerance in colorectal cancer. Cancer Immunol Immunother 61, 335–342 (2012). 21913024 10.1007/s00262-011-1108-1 PMC11028813

[b17] Liu Z. Q. *et al.* Tolerogenic CX3CR1+ B cells suppress food allergy-induced intestinal inflammation in mice. Allergy 68, 1241–1248 (2013). 24033604 10.1111/all.12218

[b18] Pilat N. *et al.* T-regulatory cell treatment prevents chronic rejection of heart allografts in a murine mixed chimerism model. J Heart Lung Transplant. 33, 429–437 (2014). 24468120 10.1016/j.healun.2013.11.004 PMC3991417

[b19] Bhuyan Z. A. *et al.* CD98hc regulates the development of experimental colitis by controlling effector and regulatory CD4+ T cells. Biochem Biophys Res Commun. 444, 628–633 (2014). 24491544 10.1016/j.bbrc.2014.01.144

[b20] Coelho V., Saitovitch D., Kalil J. & Silva H. M. Rethinking the multiple roles of B cells in organ transplantation. Curr Opin Organ Transplant. 18, 13–21 (2013). 23254702 10.1097/MOT.0b013e32835c8043

[b21] Parekh V. V. *et al.* B cells activated by lipopolysaccharide, but not by anti-Ig and anti-CD40 antibody, induce anergy in CD8+ T cells: role of TGF-beta 1. J Immunol. 170, 5897–911 (2003). 12794116 10.4049/jimmunol.170.12.5897

[b22] Munger J. S. *et al.* The integrin alpha v beta 6 binds and activates latent TGF beta 1: a mechanism for regulating pulmonary inflammation and fibrosis. Cell. 96, 319–28 (1999). 10025398 10.1016/s0092-8674(00)80545-0

[b23] Roomi M. W., Kalinovsky T., Monterrey J., Rath M. & Niedzwiecki A. In vitro modulation of MMP-2 and MMP-9 in adult human sarcoma cell lines by cytokines, inducers and inhibitors. Int J Oncol. 43, 1787–98 (2013). 24085323 10.3892/ijo.2013.2113 PMC3834263

[b24] Huang Y. R. *et al.* Ureic clearance granule, alleviates renal dysfunction and tubulointerstitial fibrosis by promoting extracellular matrix degradation in renal failure rats, compared with enalapril. J Ethnopharmacol. 155, 1541–52 (2014). 25087615 10.1016/j.jep.2014.07.048

[b25] Yang S. B. *et al.* Staphylococcal enterotoxin B-derived haptens promote sensitization. Cell Mol Immunol 10, 78–83 (2013). 22941409 10.1038/cmi.2012.32 PMC4003168

